# Sociodemographic Correlates of Organized Sports Participation in a Sample of Middle School Students in China

**DOI:** 10.3389/fpubh.2021.730555

**Published:** 2021-11-18

**Authors:** Tao Ren, Jin Yan, Qiang Sun

**Affiliations:** ^1^Centre for Science and Experiment, Nanjing Sport Institute, Nanjing, China; ^2^Priority Research Centre for Physical Activity and Nutrition, School of Education, University of Newcastle, Callaghan, NSW, Australia; ^3^Key Laboratory of Sports Training and Rehabilitation of Jiangsu Province, Nanjing, China

**Keywords:** correlates, physical activity, organized sport, adolescents, China

## Abstract

**Background:** Organized sport participation (OSP) is considered as one method with the potential to increase overall physical activity (PA) levels in young people. It is essential to understand the correlates of OSP to inform future PA interventions.

**Purpose:** This study aimed to explore the sociodemographic correlates of OSP among middle school students from the Nanjing City of China.

**Methods:** A total of 7,097 adolescents (50.1% girls) aged 12–15 years from Nanjing, China, were recruited in this survey. Self-reported data on sex, grade, race, residence areas, proficient sport skills, and parental highest education were obtained. OSP was assessed by the question asked in the questionnaire on whether participants were involved in any “sports club or team” with the binary answer options of “yes” and “no,” for statistical analysis purposes. Generalized linear models were used to determine the correlates of OSP.

**Results:** Only 16.6% reported participating in any organized sport over the past whole year, while boys (OR = 1.34, 95% CI: 1.18–1.53) and 7th graders (OR = 1.40, 95% CI: 1.18–1.65) were more likely to participate in organized sport. Adolescents being Han ethnicity were less likely to either participate in organized sport (OR = 0.60, 95% CI: 0.40–0.92), or masterless (one or two) proficient sport skills [OR (one) = 0.27, 95%CI: 0.20–0.37; OR (two) = 0.43, 95% CI: 0.36–0.50]. Besides, both residence area and parental highest education were not significantly associated with OSP among the participating adolescents.

**Conclusion:** The current study confirmed that only one-sixth of adolescents participate in the organized sport over the past year. At-risk population subgroups include girls, older adolescents, being Han ethnicity, and those proficient in fewer sport skills. Sex, grades, race, and proficient sport skills were significant correlates of OSP. School, community, and families need to provide more resources and support for disadvantaged populations in OSP.

## Introduction

The high prevalence of physical inactivity among adolescents has caused a challenging issue relevant to public health. Studies have displayed that physical inactivity would impose a negative influence on youth's physical health, cognitive outcomes, and well-being (1–3). World Health Organization has recommended that children and adolescents should do at least an average of 60 min per day of moderate- to vigorous-intensity physical activity (MVPA) across the week (4, 5). A representative national survey from China has shown that only 13% of young people participated in MVPA for 60 min and longer every day in the past 7 days (6). Organized sport participation (OSP) has been regarded as an effective medium for promoting physical activity (PA) (7–10). For example, a study has revealed that children involved in organized sport one time a week had doubled the chance of meeting the recommendations of PA than those without OSP. As such, the significance of OSP has drawn great attention from health practitioners and policymakers. Furthermore, the consensus has prevailed among public health that PA participation could not only promote individuals' physical health but also improve their psychological and social well-being (11, 12). Therefore, it is essential to explore the correlates of OSP in promoting PA participation among children and adolescents.

According to Global Matrix 3.0, Physical Activity Report Card Grades for Children and Youth, the overall prevalence of OSP among 49 countries and regions was 47–53% (13). In Canada, Sweden, Australia, and other western countries, more than 60% of youth have participated in organized sport (13). However, only 20% of Chinese children and adolescents aged 9–17 years had taken part in the organized sport over the past 12 months (6). To improve the OSP level and maximize its health benefits, it is necessary to examine the correlates of OSP. In general, boys have reported more participation in organized sports than girls (7, 14, 15). Previous studies have found that there was a serious decline in OSP during early adolescence (16–19), while other studies have indicated that the decline of OSP in different age groups was not significant (17). Similarly, among those living in rural or urban areas, the prevalence of OSP has presented inconsistent residence differences. The reason for this conclusion may be associated with specific social contexts typical of different countries. Furthermore, the economic status of the family is another important factor affecting sports participation. Since many sports activities require funding for equipment, clothing, and venues, families with low income are deprived of the opportunities to participate in certain sports (18, 20, 21). The parental education level was also a key index of socioeconomic status, but the associations between parental education level and OSP were uncertain (22). In summary, the associations between sociodemographic factors and OSP needed to be further explored.

Although there have been plenty of studies on the youth OSP, most of them were conducted in western countries where sociocultural contexts differed from those in China (23). Hence, it is unclear whether these findings (19, 24, 25) drawn from high-income countries are generalizable to children and adolescents in middle- and upper-income countries. Besides, further investigations are needed on the inconsistent findings. Given the importance of exploring correlates of OSP and the current gaps in the literature, this study aimed to investigate the sociodemographic correlates of OSP among middle school students from the Nanjing City of China.

## Methods

### Study Design and Participants

This was a cross-sectional study that took place over the period from November to December 2019. A stratified sampling and a multistage cluster sampling were used in this study. A selection of communities/towns was made with stratification by socioeconomic status within each urban and rural stratum. First, four districts were recruited from Nanjing. Second, two communities in an urban area and one town in a rural area were selected from each of the four districts. Third, two public junior middle schools were selected from all schools in each community and town. Finally, among these schools, two classes from each grade (Grades 6–8) were recruited. There are about 55 students in each class. The study recruited a total of 7,356 students to complete the web-based questionnaire survey. All the adolescents involved in the study, along with their guardians and parents, were informed of the voluntary nature of the study upon recruitment. Informed written consent was obtained from the participants and their parents or guardians before the subjects entered into the study according to Helsinki Declaration. In response, 7,097 participants (96.4% response rate) completed the survey, from which all data have been obtained and analyzed anonymously. The procedure and protocol involved in the current study are adopted based on the approval from the Institutional Review Board of Nanjing Sport Institute, while heads and teaching staff of the schools included in the current study provided cooperation and helped obtain permission to conduct the study.

### Measures

#### Independent Variables (Possible Sociodemographic Factors)

In this study, we selected the following sociodemographic factors as potential explanatory variables associated with OSP, which included sex (male or female), grade (years in school), ethnicity (whether the students are Han or belonging to other ethnic groups), residence (whether the students live in the urban or rural areas), number of proficient sport skills (1, 2, 3, and more), parental highest education (elementary school or below; middle school; high school; vocational school; university bachelor; a master; or higher). The inquiry of these independent variables was formed as questions in the survey, and all participants were asked to complete these questions upon returning the questionnaires.

#### Dependent Variables (Participation in Organized Sport)

Based on the answers to the questions on the questionnaire, participation in the organized sports can be evaluated. Participation in organized sport was measured by the following item: “Do you join any sports teams or clubs over the past year?” The respondents answer “yes” or “no” to the question. So that the purpose of statistical analysis can be met. In the questionnaire, OSP was defined as any participation in sports clubs or teams, which may include, but are not limited to, badminton club, basketball team, tennis club, running team, and volleyball club. Similar questionnaire questions were found in previous literature and studies, showing acceptable reliability. For instance, a study conducted by the American Youth Risk Behavior Survey, which investigated the test–retest reliability for 2 weeks based on a comparable question to ours, showing that grade 7 and 10 students could report their participation in organized sports activities reliably during the past year (*r* = 0.84) (26).

### Statistical Analysis

All statistical analyses in this study were performed by SPSS, version 24 (IBM, Corp. Armonk, NY, United States) in this study. First, descriptive statistics were used to demonstrate the proportion of participation (did or did not participate) in OSP among the students. Then, chi-square tests were used to determine differences between the prevalence by sociodemographic (sex, grade, race, residence, sports skills, and parental highest education) and OSP. Finally, the generalized linear models were used to investigate the associations between the selected sociodemographic factors and OSP. The statistical significance was set up as *p* < 0.05, with *p* being two-sided.

## Results

The sociodemographic characteristics of the total sample in this study are shown in Table 1. A total of 7,097 adolescents aged 12–15 years were included in the analysis. The percentage of boys and girls was 49.9 and 50.1%, respectively. The proportion of the adolescents of the three grades were 40.8% (seventh graders), 35.1% (eighth graders), and 24.1% (ninth graders), respectively. The participants were mainly of Han ethnicity (98.2%) and about three-quarters of all participants lived in urban areas (75.8%). More than half of the participants reported that they were proficient in three or more sports skills (62.8%). Only 9.5 and 27.7% of participants reported that they were proficient in one sport skill and two sport skills, respectively. As for parental highest education level, about 92.3% of the guardians reported middle school to university bachelor (19.1% of middle school, 26.7% of high school, 23.0% of occupational college, and 23.5% university bachelor).

**Table 1:** Sociodemographic features of the general sample.

		**Total**	
		***n***	**%**
Total		7,097	100.0
Sex			
	Boy	3,538	49.9
	Girl	3,559	50.1
Grade			
	7th	2,898	40.8
	8th	2,487	35.0
	9th	1,712	24.1
Ethnicity			
	Han	6,970	98.2
	Minority	127	1.8
Residence area			
	Urban	5,383	75.8
	Rural	1,714	24.2
Number of proficient sport skills			
	1	672	9.5
	2	1,965	27.7
	3 and more	4,460	62.8
Parental highest education			
	Primary school or below	168	2.4
	Middle school	1,358	19.1
	High school	1,894	26.7
	Occupational college	1,631	23.0
	University bachelor	1,668	23.5
	Master or higher	378	5.3

The prevalence of OSP by sociodemographic variables is shown in Table 2. Only 16.6% of adolescents reported involving in a sports club or team, while most of the participants (83.4%) did not take part in any organized sport over the past year. With the exceptions of residence and parental highest education, significant differences between the prevalence of OSP were observed across the remaining sociodemographic factors (*p* < 0.05 for all other comparisons).

**Table 2:** Prevalence of participation in organized sports activities by sociodemographic variables.

	**Yes (*****n*** **= 1,177)**		**No (*****n*** **= 5,920)**		***Chi-square***	***p-value***
	***n***	**%**	***n***	**%**		
**Sex**						
Boy	681	57.9	2,857	48.3	36.16	0.00
Girl	496	42.1	3,063	51.7		
**Grade**						
7	551	46.8	2,347	39.6	22.13	0.00
8	384	32.6	2,103	35.5		
9	242	20.6	1,470	24.8		
**Race**						
Han	1,146	97.4	5,824	98.4	5.72	0.02
Minority	31	2.6	96	1.6		
**Residence area**						
Urban	889	75.5	4,494	75.9	0.08	0.78
Rural	288	24.5	1,426	24.1		
**Number of proficient sport skills**						
1	43	3.7	629	10.6	181.05	0.00
2	193	16.4	1,772	29.9		
3 and more	941	79.9	3,519	59.4		
**Parental highest education**						
Primary school and below	36	3.1	132	2.2	5.92	0.31
Middle school	237	20.1	1,121	18.9		
High school	316	26.8	1,578	26.7		
Occupational college	249	21.2	1,382	23.3		
University bachelor	273	23.2	1,395	23.6		
Master or higher	66	5.6	312	5.3		

The associations between sociodemographic factors and OSP are shown in Table 3. Compared with girls, boys displayed a greater odds of joining organized sport (OR = 1.34, 95% CI: 1.18–1.53). The odds ratios (ORs) for participating in organized sport among seventh graders (OR = 1.40, 95% CI: 1.18–1.65) were significantly higher than those among ninth graders. However, compared with ninth graders, the odds of OSP among eighth grades were not statistically significant. Adolescents being Han ethnicity were less likely to participate in organized sport (OR = 0.60, 95% CI: 0.40–0.92) than those minority youth. Moreover, the number of proficient in sport skills was positively associated with the increased odds of participating in organized sport. Comparing with those proficient in three or more sports skills, adolescents reporting proficiency in one sport skill and two sport skills were less likely to join in organized sport (OR = 0.27, 95% CI: 0.20–0.37 and OR = 0.43, 95% CI: 0.36–0.50, respectively). Furthermore, residence area and parental highest education level were not significantly associated with OSP among adolescents aged 12–15 years.

**Table 3:** Odds ratio and 95% CI for socioeconomic factors concerning the participation in organized sport.

		**OR**	**95% CI**		**Sig**
Sex					
	Boy	1.34	1.18	1.53	0.000
	Girl	REF			
Grade					
	7th	1.40	1.18	1.65	0.000
	8th	1.13	0.94	1.34	0.193
	9th	REF			
Race					
	Han	0.60	0.40	0.92	0.018
	Minority	REF			.
Residence area					
	Urban	1.00	0.86	1.17	0.989
	Rural	REF			
Number of proficient sport skills					
	1	0.27	0.20	0.37	0.000
	2	0.43	0.36	0.50	0.000
	3	REF			.
Parental highest education					
	Primary school and below	1.39	0.87	2.22	0.166
	Middle school	1.06	0.78	1.44	0.721
	High school	1.02	0.76	1.38	0.877
	Occupational college	0.89	0.66	1.21	0.451
	University bachelor	0.96	0.71	1.29	0.780
	Master or higher	REF			

*REF, reference group*.

## Discussion

Only one-sixth of Nanjing adolescents aged 12–15 years participated in a sports club or team over the past year, which is much lower than their counterparts in the developed countries (13). Similarly, the prevalence of OSP among Nanjing adolescents was also lower than the participation rate of national adolescents (6). Besides, this study mainly found that boys—being seventh graders, minorities, and those proficient in more sports skills—were more likely to participate in organized sport among Chinese secondary school students. However, there were no significant associations between residence area, parental highest education, and participation in organized sport.

Consistent with previous studies (7, 14, 27), our study found that boys were more likely to be involved in sport participation than girls. Results from Shanghai's (China) 2016 Report Card on Physical Activity showed that the percentage of girls (aged from 6 to 18 years) joining organized programs or sports was only 12%, which was lower than that of boys (17%) (28). The prevalence data reflected that girls usually perceived great barriers in participating in sports activities. One of the important reasons is gender disparity (8). A prior study indicated that girls' sports participation is lower than that of boys (29). In a limited sports environment or resources, boys usually dominated the spaces and facilities for sports participation, while girls may be isolated and sometimes excluded (8, 30). As such, it is necessary to guarantee opportunities and resources of sport participation in the school environment. Besides, considering the different social roles or calling it gender stereotypes (30), boys display a greater possibility of joining in sports activities, while girls usually participate in more leisure activities and individual artistic activities (7, 8). Therefore, it is significant to build up positive female images in the sporting context where the existing stereotypes and inspired sport participation (especially for girls) could be weakened.

Previous results from the China 2018 Report Card on Physical Activity demonstrated that OSP in secondary school (22.1%) was significantly higher than in upper secondary school (15.7%) (6). This decline of OSP among school stages was also found in different grades in junior middle school. Only seventh graders showed statistically significantly higher odds of participation in organized sport compared with ninth graders. Besides, there was no significant difference in OSP level between eighth and ninth graders. In a 2-year longitudinal cohort study, a significant reduction in sports participation was also found among grade 6 to grade 8 students (aged from 11 to 13 years) and from grade 8 to grade 10 students (aged from 13 to 15 years) (16). Considering the wide academic pressure gap, the significant difference between seventh graders and highest graders was reasonable among Chinese secondary students. Due to heavy academic pressure, adolescents in the highest grades facing high school academic examinations usually give up most of their leisure time as well as sports participation (6). This kind of unhealthy lifestyle was uncommon in lower grades. However, parents, teachers, and other practitioners did not fully realize the fact that sports participation was a positive predictor of academic achievement (31). A key point of developing a healthy lifestyle is to achieve a balance between academic and sports participation, especially for higher graders.

Adolescents of the Han race were less likely to participate in sports activities in our study compared to those who were of minority ethnicities. Differences in behavior patterns between the minority and majority ethnicities are usually neglected in China. Our study is one of the few studies that examined associations between ethnicity and sports participation among Chinese adolescents. Previous studies indicated that minorities (Latinos) generally had higher perceived constraints (including accessibility, knowledge, partners) that limited adolescent sport participation than majority ethnicity (Caucasian) (15). However, another study showed that minorities have a slightly higher prevalence than the majority when adjusting for the socioeconomic status (32), which was consistent with our finding to some extent. Considering composition differences in ethnicities, the evidence from other countries could not be completely generalized to adolescents in China. Cultural and geographical factors are possibly important predictors that lead to differences in sport participation for Chinese adolescents (33). Compared with minority ethnicity parents, Han ethnicity parents are given high expectations of academic success and put higher academic pressure on their children. While there are a larger number of minorities with spontaneously and regularly OSP, such as climb mountains, folk dance, dragon dance, and wrestling, there may have less leisure time participating in sports activities among Han ethnicities. In general, the significant finding was high odds of sport participation among minorities. It may be contributed to explore the factors of sports participation in students.

Sports skills may be positively correlated with sport participation in adolescents. In other words, lack of sports skills was considered a significant barrier to sports participation. This viewpoint was verified by some qualitative studies (29, 30, 34, 35). One quantitative study explained that participants who grasped at least one sport skill display greater likeliness three times higher than non-active adolescents. Another study explained that students who joined in low to moderate PA have greater likeliness to report that they joined in one or more sports teams twice higher than sedentary individuals (8). Besides, among 50,020 Shanghai children and adolescents, Zhu and co-authors found that the odds of meeting the PA guidelines displayed a positive association with the number of reported sport skill proficiency (36). From the practical significance, proficiency in more sports skills can be flexibly tailed to multiple sports contexts. Meanwhile, more enjoyment and achievement gained from sports participation was also based on proficiency in more sports skills. Therefore, it should be noted that coaches, physical education, and parents should give more financial and emotional support for various motor skills learning processes. More importantly, schools and communities may need to employ more specialists who were proficient in more sports skills.

Based on the current study, there was no significant relationship between the parental education level, residence, and sport participation in adolescents. The associations between socioeconomic status and PA were inconsistent (15, 16, 23, 37). Most studies reported that socioeconomic gradients were parallel to gradients in adolescents' sport participation (15, 16, 23). A 2-year longitudinal study found that increasing socioeconomic status was a predictor of starting or persisting sports among 13–15 adolescents (16). However, evidence retrieved from a longitudinal school study in Denmark indicated that income had no direct impact on organized sport due to the comparatively low cost of organized sport (37). Furthermore, it should be noted that income had no direct impact but showed indirect influence on OSP through the involvement of parents (37, 38). In other words, adolescents from low-income families can have a higher likelihood of participating in the organized sport when their parents are usually involved in sport with them. As for parental education level, previous studies demonstrated that adolescents have more potential opportunities to participate in activities in lower parental education-level families, compared to higher education-level families (39). Therefore, according to our findings and previous evidence, one approach was to encourage parents to pay attention to their children in different aspects. From the perspective of policy, involving sport in organized PA might be regarded as a way to level the playing field. Furthermore, future studies need to explore the role of parental involvement played in the associations between education level and OSP.

Several limitations should be acknowledged in this study. First, the prevalence of OSP was measured by a self-report questionnaire, which may lead to recall bias to items. Second, the number of proficient sport skills measured by self-perceived may be inaccurate due to adolescents' different definitions of skill proficiency. Third, several variables may relate to adolescents' OSP that have not been discussed in the present study (e.g., family income, weight status).

## Conclusion and Implications

To conclude, only one-sixth of adolescents participated in the organized sport over the past year, which puts them at risk. At-risk population subgroups include girls, older adolescents, those of Han ethnicity, and those less proficient in sport skills. Considering the significant health benefits of OSP, our findings support the call for public health agencies to design specific interventions for population-level organized sports participation and to consider at-risk groups. First, public health agencies need to provide more opportunities for adolescents, especially girls, to participate in organized sport in schools or communities. Second, we recommend that adolescents organized sport with parental support, especially where parents act as role models in activities to encourage the participation of adolescents. Third, it is necessary to account for the academic pressure on older adolescents and encourage them to participate in organized sport by allowing them enough time to exercise as part of their study schedule.

## Data Availability Statement

The raw data supporting the conclusions of this article will be made available by the authors, without undue reservation.

## Author Contributions

TR and JY involved in the study design and drafted the manuscript. TR and QS performed data analysis. JY and QS involved in the revision of the manuscript. All authors contributed to the article and approved the submitted version.

## Funding

This work was supported by Study on the Change Characteristics and Limiting Factors of Sensitive Qualities of Young Athletes in Different Developmental Stages (Grant NO:SYS202104).

## Conflict of Interest

The authors declare that the research was conducted in the absence of any commercial or financial relationships that could be construed as a potential conflict of interest.

## Publisher's Note

All claims expressed in this article are solely those of the authors and do not necessarily represent those of their affiliated organizations, or those of the publisher, the editors and the reviewers. Any product that may be evaluated in this article, or claim that may be made by its manufacturer, is not guaranteed or endorsed by the publisher.
